# Severity and Its Contributing Factors in Patients With Vestibular Migraine: A Cohort Study

**DOI:** 10.3389/fneur.2020.595328

**Published:** 2020-12-16

**Authors:** Wei Liu, Hongli Dong, Le Yang, Hongru Zhao, Wanli Dong, Yi Yang

**Affiliations:** ^1^Departments of Neurology, The First Affiliated Hospital of Soochow University, Suzhou, China; ^2^Department of Neurology, Suzhou TCM Hospital Affiliated to Nanjing University of Chinese Medicine, Suzhou, China; ^3^School of Public Health, Fujian Medical University, Fuzhou, China

**Keywords:** vestibular migraine, severity, clinical presentation, contributing factors, circadian variations

## Abstract

**Objective:** As a recently defined disease entity, vestibular migraine (VM) is a variant of migraine with broad spectrum of manifestations. We evaluated a prospective cohort of patients with VM in two centers to assess severity of VM attacks and investigate its contributing factors in patients with VM.

**Methods:** Adult participants with the diagnosis of VM or probable VM were enrolled according to the 2012 International Headache Society-Bárány Society Criteria. Every outpatient was followed up for 6 months to record the occurrence of VM attacks. Clinical data such as age, sex, number of VM attacks, severity on the visual analog scale, and lipid intake were collected and analyzed. Generalized Anxiety Disorder-7, Patient Health Questionnaire-9, Horne and Ostberg Morningness-Eveningness Questionnaires, and Pittsburgh Sleep Quality Index were also administered to find contributing factors.

**Results:** During a 6-month clinical follow-up, 313 VM attack were reported. According to the Visual Analog Scale, the patients were divided into two groups. Then univariate and multivariable analyses were conducted. Among the risk factors, duration of illness (adjusted OR, 1.041; 95% CI, 1.010–1.073; *P* = 0.009), time of onset: 00:00:00–12:00:00 (adjusted OR, 3.961; 95% CI, 1.966–7.979; *P* < 0.001) and PSQI scores (adjusted OR, 1.086; 95% CI, 1.002–1.178; *P* = 0.046) were significantly associated with the severity of VM attack assessed by VAS.

**Conclusion:** The data suggest that patients tended to experienced more severe VM attacks in early hours of a day, especially for those sufferers with longer duration of illness or poor sleep quality. Targeted management of such factors is required to reduce the severity of attacks.

## Introduction

Vestibular migraine (VM), as the most common cause of episodic vertigo, is a disease with significant impact on the quality of life for those afflicted (1, 2). The diagnosis and treatment of VM are challenged by its broad manifestation spectrum, uncertain triggers, and complex comorbidities (3).

For VM patients, they may have very different onset time or clinical manifestations in different attacks (3, 4). The evaluation of these patients' subjective dysfunction and psychological status, which causes some considerable difficulties in daily life, is useful to further understand the disease process (5). Meanwhile, various triggers are frequently reported by VM patients, but its contributing factors and pathophysiology are still uncertain (4, 6). To explore the self-awareness and related clinical manifestations of VM patients is helpful to formulate more specific intervention measures.

In this study, we explored the demographic and clinical characteristics of VM patients with a 6-month follow-up. At the same time, we assessed the severity of VM attacks and investigated its related risk factors.

## Methods

### Study Population

Participants were recruited to meet the 2012 International Headache Society-Bárány Society Criteria, which were included in the third version of the International Classification of Headache Disorder for VM or possible VM (7, 8) from September 2014 to May 2018 at the neurology outpatient clinics of the First Affiliated Hospital of Soochow University and Suzhou TCM Hospital Affiliated to Nanjing University of Chinese Medicine (as seen in Table 1). The diagnoses were made by two senior neurologists and one senior otolaryngologist.

**Table 1:** Diagnostic criteria of vestibular migraine from the 2012 International Headache Society and Bárány Society.

**Vestibular migraine**
A. At least five episodes of vestibular symptoms^+^ of moderate to severe intensity lasting 5 min−72 h
B. Current or previous history of migraine +/ aura according to the International Classification of Headache Disorders (ICHD).
C. One or more migraine features^++^ with at least 50% of the vestibular episodes.
D. Not better accounted for by another vestibular or ICHD diagnosis.
**Probable vestibular migraine**
A. At least 5 episodes with vestibular symptoms^+^ of moderate to severe intensity lasting 5 min−72 h.
B. Only one of the criteria B and C for vestibular migraine is fulfilled.
C. Not better accounted for by another vestibular or ICHD diagnosis.

*Vestibular symptoms^+^: Spontaneous vertigo (Internal vs. External); positional vertigo; visually-induced vertigo; head motion-induced vertigo; head motion-induced dizziness with nausea*.

*Migraine features^++^: Visual aura, photophobia, phonophobia, and/or headache with at least two distinct features (e.g., one-sided location, moderate to severe pain intensity, aggravation by routine physical activity, pulsating quality)*.

The initial sample included 675 persons. The exclusion criteria were as follows: (1) <18 years old; (2) during the follow-up period, no attack or loss of follow-up was reported; (3) inability to complete questionnaires within 72 h after attack; (4) headache or vestibular symptoms attributed to secondary causes; (5) long-term users (more than 2 weeks) of migraine-suppressing drugs such as low-dose tricyclic antidepressants, calcium channel blockers, or beta-blockers; (6) long-term users (more than 2 weeks) of hypnotics; (7) users of oral contraceptives; (8) history of drug abuse; (9) history of head trauma, otorhinolaryngology surgery, or intracranial infection; (10) shift work.

Only 351 patients with VM or probable VM were included in the present study. A 6-month clinical follow-up was performed. Nine patients were lost for 342/351 (97.4%) of the patients, and 35 patients did not report any attack during the follow-up. Finally, 307 participants (241 definite VM and 66 probable VM) formed the basis of this report. All patients received the same advice, including sleep hygiene, physical exercise, identifying and blocking the triggers of migraine.

### Outcome Variable

The main outcome measures were VM episodes reported by patients during follow-up (from June 01, 2019 until the end of the follow-up on December 31, 2019). The recruited patients were followed up for inter-ictal phase by telephone and emails. If a participant reported any form of seizure, she/he was interviewed by two neurologists and record their characteristics. All self-reported attack cases and diagnostic dates were verified using participants' hospital records and information collected from their physicians. Participants were allowed to take acute medications according to their medical conditions. We recorded and classified the sensation descriptions of patients during VM episodes according to the definitions of the Committee for Classification of Vestibular Disorders of the Bárány Society in 2012 and “Classification of Vestibular Symptoms” of the Bárány Society in 2009 (8, 9).

### Evaluation of Severity

Visual analog scale (VAS) was used to assess patients' subjective perception of severity, including but not limited to vestibular symptoms, headache symptoms, visual symptoms, and ictal aural symptoms (VAS was classified as follows: 0–3, mild; 4–6, moderate; and 7–10, severe) (10, 11). All participants were asked to complete the VAS scale with the assistance of a neurologist within 6 h of the self-report. Each participant was asked to answer three times, with an hour interval between each inquiry. The final VAS score was the average value of these three times.

### Baseline Information and Measurement of the Covariates

Each participant took part in a face-to-face interview by trained staff to collect baseline data. Demographic information, lifestyles (drinking or caffeine intake), family history, and health history (e.g., self-reported or physician diagnosed motion sickness) were gathered. Family history was defined as the reporting of similar vestibular symptoms by blood related family members (lasting more than 5 min to a few days) with no clear diagnosis or with migraine. Lctal aural symptoms were defined as tinnitus, ear fullness/pressure, and/or muffled hearing (these symptoms were binaural or monaural) (12). Visual symptoms were defined as visual aura, visual blurring, eye strain, and/or visual distortions (these symptoms were binocular or monocular) (12).

Those participants who fulfilled the inclusion criteria completed the following questionnaires in 72 h after attack: (1) a Chinese translation of Horne and Ostberg Morningness-Eveningness Questionnaires (MEQ) was used to assess the participants' chronotype (13); (2) the Generalized Anxiety Disorder-7 (GAD-7) was used to estimate the patient's anxiety level (14); (3) the Patient Health Questionnaire-9 (PHQ-9) was used to estimate the patient's level of depression (15); (4) the Chinese version of the Pittsburgh Sleep Quality Index (CPSQI) was used to assess sleep quality (16); the Chinese version of PSQI also showed high reliability (Cronbach'sα coefficient = 0.83), and the best cut-off point was 5 (16); (5) a validated semiquantitative food frequency questionnaire was used to examined recent food intake patterns (on average over the previous 2 week; high-lipid diet: the total lipid intake was >100 g daily; alcohol or caffeine consumption: take in weekly or more often) (17, 18).

### Data Analyses

Continuous variables were analyzed as mean and standard deviation or the median and interquartile range while categorical variables were analyzed as frequency and percentage, properly. Student's *t*-test, Mann-Whitney U test or Chi-square test was used to assessed differences among variables. Pearson's correlation coefficients were calculated to assess the relationship between the variables. Logistic regression analysis was used to find risk factors associated with severity of attack in patients with VM after adjusting for other variables selected from univariate analyses. Therefore, no priori power analysis was conducted to calculate sample size. The level of significance for these descriptive comparisons was established at 0.05 for two-sided hypothesis testing. Statistical analysis was performed in SPSS 25.0 and R programming language (3.6.3).

## Results

### Participants and Their Demographics and Characteristics

**Figure 1:**
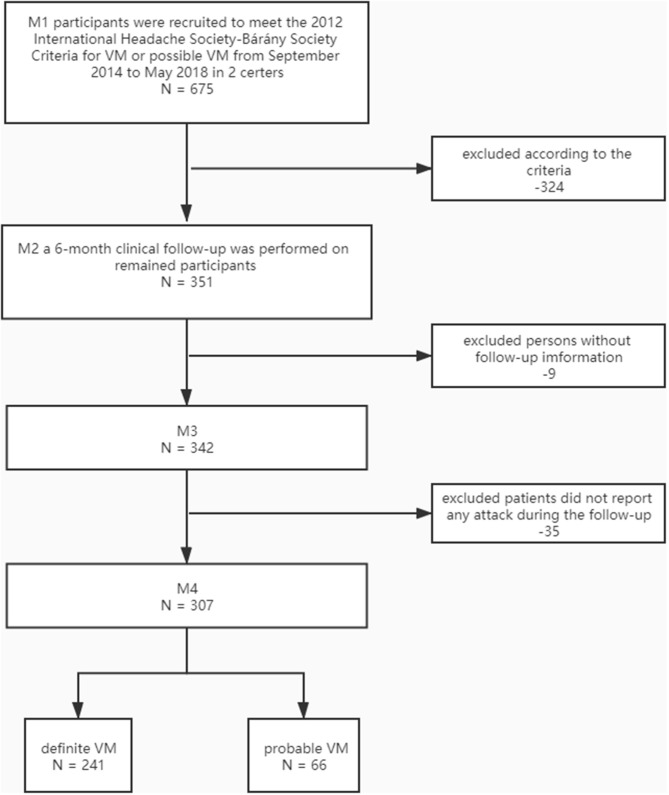
Flow diagram of participants' selection.

A total of 307 patients with VM were included in our study; only four (1.3%) of them reported two attacks, two (0.6%) of them reported three attacks. As the number of participants with multiple attacks was very small, no useful separate analysis could be made. Therefore, we identified their first attacks during the follow-up period to further analysis. Patient selection is illustrated in Figure 1.

Among our subjects, just 28.00% were males (221 women, 86 men); the age was 42.0 (35.0, 52.0) years old; the duration of illness was 4.0 (1.0, 9.0) years; 155 patients (50.50%) reported a family history of similar episodic vestibular symptoms. Most sufferers (73.60%) experienced VM attacks in early hours and length of spell was 2.00 (0.50, 8.00) hours. Few patients reported alcohol consumption (9.40%) or caffeine consumption (3.60%). Patient characteristics were shown in Table 2.

**Table 2:** The characteristics of patients with VM.

**Characteristics**	**Patients (*N* = 307)**
Age, years, median (IQR)	42.00 (35.00, 52.00)
Male, *n* (%)	86 (28.00)
Age of first onset, median (IQR)	36.00 (30.00, 44.00)
Duration of illness, years, median (IQR)	4.00 (1.00, 9.00)
History of motion sickness, *n* (%)	126 (41.00)
Family history, *n* (%)	155 (50.50)
High-fat diet, *n* (%)	12 (3.90)
Alcohol consumption, *n* (%)	29 (9.40)
Caffeine consumption, *n* (%)	11 (3.60)
Time of onset, *n* (%) 00:00:00–12:00:00	226 (73.60)
12:00:00–24:00:00	81 (26.40)
Length of spell, hours, median (IQR)	2.00 (0.50, 8.00)
Ictal aural symptoms, *n* (%)	47 (15.30)
Visual symptoms, *n* (%)	48 (15.60)
VAS,median (IQR)	6.00 (5.00, 8.00)
GAD-7, median (IQR)	11.00 (8.00, 14.00)
PHQ-9, median (IQR)	9.00 (7.00, 12.00)
PSQI, median (IQR)	8.00 (6.00, 10.00)
MEQ, median (IQR)	56.00 (50.00, 63.00)

*VAS, Visual Analogs Scale; GAD-7, Generalized Anxiety Disorder-7; PHQ-9, Patient Health Questionnaire-9; PSQI, Pittsburgh Sleep Quality Index. MEQ, Horne and Ostberg Morningness-Eveningness Questionnaires*.

### Comparison of Demographic, Clinical and Psychometric Characteristics in VM Patients

**Figure 2:**
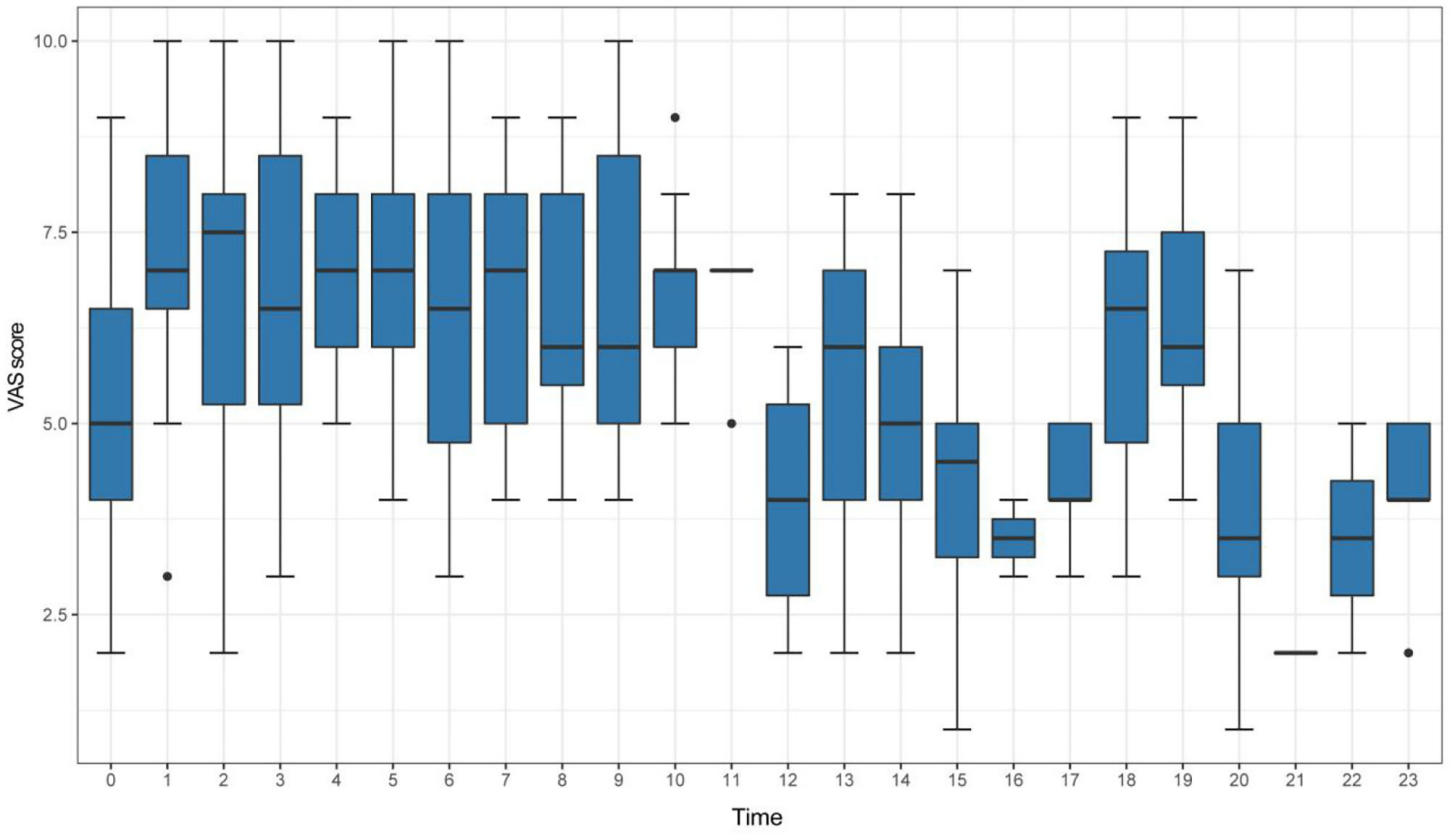
Box plots for time and VAS scores in VM patients.

According to the VAS scores of identified attacks, the VM patients were divided into two groups: the high VAS group with 137 patients (VAS ≥ 7) and the low VAS group with 170 patients (VAS < 7). The box plots in Figure 2 and Table 3 summarizes these data.

**Table 3:** Comparison of demographic, clinical and psychometric characteristics between groups of VM patients with high and low VAS.

**Characteristics**	**VAS ≥ 7 (*N* = 137)**	**VAS < 7 (*N* = 170)**	***P***
Age, years, median (IQR)	44.00 (36.00, 56.50)	41.00 (36.00, 49.00)	0.041
Male, *n* (%)	40 (29.2)	46 (27.1)	0.678
Age of first onset, median (IQR)	36.00 (29.50, 45.00)	36.50 (30.75, 42.00)	0.665
Duration of illness, years, median (IQR)	5.00 (2.00,11.00)	3.00 (1.00, 7.00)	0.023
History of motion sickness, *n* (%)	59 (43.1)	67 (39.4)	0.518
Family history, *n* (%)	70 (51.1)	85 (50.0)	0.849
Time of onset, *n* (%) 00:00:00–12:00:00	121 (88.3)	105 (61.8)	<0.001
12:00:00–24:00:00	16 (11.7)	65 (38.2)	
Length of spell, hours, median (IQR)	3.00 (0.50, 3.00)	2.00 (0.33, 15.50)	0.303
Ictal aural symptoms, *n* (%)	22 (16.1)	25 (14.7)	0.744
Visual symptoms, *n* (%)	17 (12.4)	31 (18.2)	0.162
Alcohol consumption, *n* (%)	14 (10.2)	15 (8.8)	0.678
Caffeine consumption, *n* (%)	6 (4.4)	5 (2.9)	0.548
High-fat diet, *n* (%)	6 (4.4)	6 (3.5)	0.702
GAD-7, median (IQR)	12.00 (9.00, 15.00)	10.00 (8.00, 13.00)	0.005
PHQ-9, median (IQR)	9.00 (7.00, 12.00)	9.00 (6.00, 11.00)	0.239
PSQI, median (IQR)	8.00 (6.00, 11.00)	8.00 (6.00, 9.00)	0.006
MEQ, median (IQR)	54.00 (48.00, 62.50)	56.00 (50.00, 64.00)	0.436

In their demographics and clinical characteristics, patients with high VAS were significantly older than those with low VAS and had longer histories of VM. And patients experienced VM attacks in early hours (00:00:00–12:00:00) had significantly higher VAS scores (*P* < 0.001). However, there was no difference in gender ratio, age of first onset, length of spell, accompanying symptoms, diet and other factors between two groups (*P* > 0.05). The scores of subjective ratings on anxiety level (GAD-7 scale) were significantly higher in the high VAS group, as was the disturbed sleep quality (PSQI scale). There were no significant differences in depressive level (PHQ-9 scale) and participants' chronotype (MEQ scale) between two groups (Table 3).

Furthermore, we calculated the correlation coefficients between time of onset, gender, age, age of first onset, duration of illness, alcohol and caffeine consumption, high-fat diet, history of motion sickness, family history, ictal aural symptoms, visual symptoms, length of spell, VAS, GAD-7, PHQ-9, PSQI, and MEQ scores and observed correlation coefficients: *r* = −0.342 between the time of VM attacks and VAS (*p* < 0.001); *r* =0.233 between the PSQI scores and VAS (*p* < 0.001); *r* = 0.175 between the GAD-7 scores and VAS (*p* < 0.001). VAS had weak but significant correlations with attack time, recent sleep quality, and anxiety (Table 4).

**Table 4:** Pearson correlation coefficients between the variables.

	**Time of onset**	**Male**	**Age**	**Age of first onset**	**Duration of illness**	**Alcohol consumption**	**Caffeine consumption**	**High-fat diet**	**History of motion sickness**	**Family history**	**Ictal aural symptoms**	**Visual symptoms**	**Length of spell**	**VAS**	**GAD-7**	**PHQ-9**	**PSQI**	**MEQ**
Time of onset	1	0.059	0.002	0.019	−0.021	0.109	−0.050	0.022	−0.160**	0.120*	0.087	0.114*	−0.003	−0.342**	−0.402**	−0.043	−0.232**	0.237**
Male	0.059	1	0.000	0.025	−0.027	0.047	0.075	−0.126*	−0.063	−0.151**	0.118*	−0.109	0.055	0.039	0.028	−0.043	0.007	0.024
Age	0.002	0.000	1	0.760**	0.547**	−0.170**	0.071	−0.189**	−0.150**	−0.054	−0.071	−0.013	0.089	0.142*	−0.052	0.128*	−0.080	0.042
Age of first onset	0.019	0.025	0.760**	1	−0.128*	−0.146*	0.131*	−0.152**	−0.107	−0.063	0.002	0.018	0.050	0.089	−0.045	0.157**	−0.067	0.030
Duration of illness	−0.021	−0.027	0.547**	−0.128*	1	−0.075	−0.061	−0.094	−0.094	−0.004	−0.114*	−0.045	0.072	0.105	−0.023	−0.007	−0.035	0.026
Alcohol consumption	0.109	0.047	−0.170**	−0.146*	−0.075	1	−0.002	0.510**	0.025	0.164**	0.079	0.076	0.146*	−0.024	0.072	0.033	−0.093	0.085
Caffeine consumption	−0.050	0.075	0.071	0.131*	−0.061	−0.002	1	0.232**	−0.054	−0.090	−0.082	−0.083	−0.008	0.007	−0.018	0.032	0.030	0.040
High-fat diet	0.022	−0.126*	−0.189**	−0.152**	−0.094	0.510**	0.232**	1	0.037	0.065	−0.039	0.098	0.020	−0.028	−0.030	−0.023	0.012	0.121*
History of motion sickness	−0.160**	−0.063	−0.150**	−0.107	−0.094	0.025	−0.054	0.037	1	0.151**	0.031	0.005	−0.031	0.047	0.074	0.122*	0.157**	−0.011
Family history	0.120*	−0.151**	−0.054	−0.063	−0.004	0.164**	−0.090	0.065	0.151**	1	0.113*	0.014	0.108	0.016	−0.034	−0.001	0.003	0.073
Ictal aural symptoms	0.087	0.118*	−0.071	0.002	−0.114*	0.079	−0.082	−0.039	0.031	0.113*	1	0.091	−0.114*	0.027	0.005	−0.010	−0.032	0.032
Visual symptoms	0.114*	−0.109	−0.013	0.018	−0.045	0.076	−0.083	0.098	0.005	0.014	0.091	1	0.003	−0.116*	0.048	−0.037	0.022	0.088
Length of spell	−0.003	0.055	0.089	0.050	0.072	0.146*	−0.008	0.020	−0.031	0.108	−0.114*	0.003	1	−0.033	0.021	−0.034	−0.100	0.058
VAS	−0.342**	0.039	0.142*	0.089	0.105	−0.024	0.007	−0.028	0.047	0.016	0.027	−0.116*	−0.033	1	0.175**	0.085	0.233**	−0.132*
GAD-7	−0.402**	0.028	−0.052	−0.045	−0.023	0.072	−0.018	−0.030	0.074	−0.034	0.005	0.048	0.021	0.175**	1	0.218**	0.129*	−0.012
PHQ-9	−0.043	−0.043	0.128*	0.157**	−0.007	0.033	0.032	−0.023	0.122*	−0.001	−0.010	−0.037	−0.034	0.085	0.218**	1	0.021	0.020
PSQI	−0.232**	0.007	−0.080	−0.067	−0.035	−0.093	0.030	0.012	0.157**	0.003	−0.032	0.022	−0.100	0.233**	0.129*	0.021	1	−0.113*
MEQ	0.237**	0.024	0.042	0.030	0.026	0.085	0.040	0.121*	−0.011	0.073	0.032	0.088	0.058	−0.132*	−0.012	0.020	−0.113*	1

* *p < 0.05*.

** *p < 0.001*.

### Contributing Factors Associated With Severity of Attacks in VM

Univariate and multivariable logistic regression analyses were used to identify the risk factors associated with severe attack in VM patients. Model were established for group of confounding factors: gender, duration of illness, history of motion sickness, family history, time of onset (00:00:00–12:00:00); ictal aural symptoms, visual symptoms, alcohol consumption, high-fat diet, PSQI, and GAD-7. From the univariate logistic analysis, duration of illness (OR, 1.033; 95% CI, 1.005–1.062; *P* = 0.019), time of onset (00:00:00–12:00:00, OR, 4.682; 95% CI, 2.553–8.584; *P* < 0.001), PSQI (OR, 1.119; 95% CI, 1.039–1.205; *P* = 0.003) and GAD-7 (OR, 1.096; 95% CI, 1.030–1.166; *P* = 0.004) were found to be significantly associated with severity of VM attack (Table 5). After adjusting for other variables selected from univariate analyses, multivariable logistic regression analysis was used to determine factors that were associated with the severity of VM attack. Our results indicated that only duration of illness (adjusted OR, 1.041; 95% CI, 1.010–1.073; *P* = 0.009), time of onset (00:00:00–12:00:00, adjusted OR, 3.961; 95% CI, 1.966–7.979; *P* < 0.001) and PSQI scores (adjusted OR, 1.086; 95% CI, 1.002–1.178; *P* = 0.046) were significantly associated with the severity of VM attack assessed by VAS. however, gender, history of motion sickness, family history, ictal aural symptoms, visual symptoms, alcohol consumption, high-fat diet and GAD-7 scores showed no association with the severity of VM attack (*P* > 0.05, Table 5).

**Table 5:** Logistic regression analysis of risk factors associated with severe attack in VM patients.

	**Univariate logistic regression analysis**		**Multivariable logistic regression analysis**	
**Factor**	**OR (95% CI)**	***p*-value**	**OR (95% CI)**	***p*-value**
Gender	1.112 (0.674–1.833)	0.678	1.054 (0.599–1.855)	0.855
History of motion sickness	1.163 (0.736–1.837)	0.518	1.020 (0.617–1.688)	0.937
Family history	1.045 (0.666–1.639)	0.849	1.031 (0.621–1.710)	0.907
Duration of illness	1.033 (1.005–1.062)	0.019	1.041 (1.010–1.073)	0.009
Time of onset: 00:00:00–12:00:00	4.682 (2.553–8.584)	<0.001	3.961 (1.966–7.979)	<0.001
Ictal aural symptoms	1.110 (0.595–2.069)	0.744	1.463 (0.727–2.945)	0.286
Visual symptoms	0.635 (0.335–1.205)	0.165	0.658 (0.326–1.327)	0.242
Alcohol consumption	1.176 (0.547–2.530)	0.678	1.458 (0.542–3.920)	0.455
High-fat diet	1.252 (0.395–3.972)	0.703	1.241 (0.286–5.396)	0.773
PSQI	1.119 (1.039–1.205)	0.003	1.086 (1.002–1.178)	0.046
GAD-7	1.096 (1.030–1.166)	0.004	1.021 (0.949–1.100)	0.574

## Discussion

In our study, the severity of the attack in VM patients was analyzed with clinical data as well as related psychological parameters. The main results are as follows: (1) patients experienced VM attacks in early hours of a day (00:00:00–12:00:00) had more severe headaches or vestibular symptoms; (2) severity of attack was associated with duration of illness and sleeping disruption. To our knowledge, this study was the first time to analyze the relationship between the time of onset and the severity of VM attack.

For most patients, VM is an episodic disorder with a combination of headache and vestibular symptoms; the duration of attacks ranges from seconds to days (4, 19). Relapses of dizziness or headache tend to be longer with VM and occur up to several times per year, compared to relapsing migraine having episodes once every few weeks (20, 21). However, the frequency of VM attacks was slightly lower in our cohort. Although no long-term migraine-suppressing drugs had been used, most participants reported only one attack during follow-up, which may be related to the subjective discomfort and self-avoidance of triggers. Thus, this issue may be further examined in VM patients in future studies.

VM may be more common in women; however, the specific proportion is quite different in different studies (5, 9, 12, 22). A recent retrospective study in Italy showed a higher probability of variability and onset with increasing age in VM patients (23). Meantime, elderly women were more often affected than elderly men (23). A systematic review of Germany reveals that disease severity has also been reported to vary over time (24). Although there were few studies on the natural history and related mechanisms in patients with VM, some reasonable hypotheses have been put forward on the fact that migraine and VM may share some common pathophysiology. This phenomenon may be related to the sharing of central and peripheral vestibular structures in older age (25). Aging affects the saccular function and its central processing in the vestibular nucleus and vestibulospinal tract (25). It is particularly true for the period from pre-school age to puberty and elderly patients (5, 12, 25). These findings are consistent with our results that VM patients with a longer history had more severe attacks. Considering that VM and migraine partly share same neural circuits, including thalamus and amygdala, hormone triggering may play an important role in age-related variability in the clinical presentation of VM (4, 12, 26). A recent study showed systemic hormone factors like hyperthyroidism could hasten age-related deterioration of saccule-related neural function (25). Among the participants, especially among the elderly, women were obviously dominant, which indirectly demonstrated this point (23).

Migraine shows circadian variation in the occurrence and clinical presentation (21). Previous studies had suggested that migraine headache characteristics, such as severe headache intensities were most frequent between 06:00 and 11:59 (21, 27). VM as a variant of migraine, also showed similar circadian rhythms in our study. About 73.6% patients experienced VM attacks in early hours of a day and the time of onset was associated with the severity of attack assessed by VAS. Meanwhile, we found poor sleep quality, as a clinical common inducement, was associated with severity of VM attack. Latest study had shown that VM patients had worse sleep quality than migraine patients and normal subjects (22). VM may cause structural sleep disorders by affecting sleep regulation centers (22). A recent study with a large sample size by Kim et al. also showed a correlation between vestibular-related diseases (including VM) and sleep disorders (28). In Furman's VM pathophysiology model, the hypothalamus is included in the network of vestibular, visceral sensory and nociceptive information (6). The circadian rhythm is controlled by a complex system of molecular regulation with a master precursor, located in the suprachiasmatic nucleus (SCN) of the anterior hypothalamus (26, 29). Severity of VM attack was influenced by early circadian attack onset and induction of sleep quality, suggested that VM patients may have a different setting of the endogenous pacemaker in the suprachiasmatic nucleus (6). The circadian clock in the SCN is a complex network of heterogeneous neuronal and glial cells (30, 31). The most obvious circadian-regulated output is sleep and wake timing (6, 30, 31). Interestingly, the possible central pathogenesis of VM involves multiple sleep related nerve nuclei like the raphe nucleus, locus coeruleus and parabrachial nucleus, and the hypothalamus also plays a key regulatory role in sleep control. The locus coeruleus, as a regulatory target of central and vestibular conduction pathways in VM during an episode, which receives signals from hypothalamus (6, 30). Neuroanatomical studies have revealed a strong interaction between the vestibular system and noxious brainstem areas such as locus coeruleus, medial and lateral vestibular nuclei, and caudal trigeminal nucleus, which provides a basis for relationship between VM and sleep disorders (30). The trigeminal nerve and vascular reflex are activated in the inner ear in patients with VM, and the vestibular nucleus and pain system signal transduction is increased, suggesting that sleep disorders and other stress states may be triggering factors of VM (22). However, it is difficult to determine the causal relationship between circadian rhythmicity, sleep homeostasis, and VM on the available evidence. We can only preliminarily infer that the attack and severity of VM may be closely related to circadian rhythm and sleep homeostasis in VM patients.

Some studies have estimated that about 50% patients with VM have concomitant psychiatric disorders, most frequently is anxiety (19, 32). Our subjects excluded dizziness caused by subjective anxiety, thus minimizing the risk of inclusion bias. At the same time, anxiety may lead to the amplification of subjective discomfort caused by VM. It is more difficult to evaluate subjective reports in patients with decreased cognitive ability. However, our data suggested that severity of VM attack was not independently related with psychological factors, including anxiety.

Our data should be interpreted with some caution due to limitations of the study. Since the participants in this study were recruited only from two clinical unit, there may have retrospective bias inherent. CPSQI was used to assess sleep quality. Although this method is not the gold standard, it is a reliable method in population-based studies. The validity of the scale was further supported by the results that subjective sleep quality, sleep latency, sleep duration, and habitual sleep efficiency sub-scores significantly correlated to corresponding measures obtained by or derived from other instruments (16). We enrolled patients with probable VM, who might develop definite VM over time as some studies have shown (33–35). Moreover, we did not assess headache and vestibular symptoms separately, considering the complex temporal patterns between them (36). The exact clinical relevance needs to be further studied.

## Conclusion

In summary, patients tended to experienced more severe VM attacks in early hours of a day, especially for those sufferers with poor sleep quality. Therefore, appropriate management of sleeping problems may reduce the severity of attack in VM patients.

## Data Availability Statement

The original contributions presented in the study are included in the article/supplementary materials, further inquiries can be directed to the corresponding author/s.

## Ethics Statement

Written informed consent was obtained from the individual(s) for the publication of any potentially identifiable images or data included in this article.

## Author Contributions

WL and YY designed the study. WL, HD, HZ, YY, and WD evaluated the subjects and collected the data. LY and YY analyzed the data. WL and HD wrote the initial draft, with YY and WD participating in revising the manuscript. All authors contributed to the article and approved the submitted version.

## Conflict of Interest

The authors declare that the research was conducted in the absence of any commercial or financial relationships that could be construed as a potential conflict of interest.
